# Mycelial dynamics in arbuscular mycorrhizal fungi

**DOI:** 10.1111/nph.70688

**Published:** 2025-10-26

**Authors:** Vasilis Kokkoris

**Affiliations:** ^1^ Amsterdam Institute for Life and Environment Vrije Universiteit Amsterdam Amsterdam 1081 HV the Netherlands

**Keywords:** allometry, anastomosis, arbuscular mycorrhizal fungi, coenocytic, hyphal networks, resilience, symbiosis

## Abstract

Arbuscular mycorrhizal fungi (AMF), similar to other filamentous fungi, develop extensive hyphal networks collectively known as mycelia. AMF mycelia are complemented by a variety of specialized structures such as spores, vesicles, and auxiliary cells, which together form integrated and functionally diverse AMF networks. AMF mycelia have long been conceptually fragmented, with research disproportionately focusing on the intraradical phase and especially on intraradical structures such as arbuscules, while usually neglecting the extraradical mycelial phase. Moreover, they are often examined from a plant‐centric perspective, where they are usually viewed as mediators of nutrient transfer to host roots. However, AMF mycelia are now increasingly recognized as a crucial component of AMF integrated networks with complex structural, physiological, and ecological dynamics. To encourage broader investigation into this underexplored domain, I synthesize both recent advances and historically overlooked findings on mycelial morphogenesis, growth strategies, resilience, cellular coordination mechanisms, and inter‐mycelial interactions. By reframing the mycelium as a single, responsive, and functionally central unit of AMF biology, I propose novel mechanisms that may shape mycelial function, highlight methodological opportunities, and suggest key open questions that must be addressed to fully understand how these hyphal networks function across scales.

**Table:** 

	Contents	
	Summary	691
I.	Arbuscular mycorrhizal fungal mycelia	691
II.	Morphology and morphogenesis	692
III.	Allometry (ERM : IRM)	700
IV.	Robustness and resilience	701
V.	Interacting mycelia	702
VI.	Content and coordination	706
VII.	Future directions	707
	Acknowledgements	708
	References	708

## I. Arbuscular mycorrhizal fungal mycelia

Arbuscular mycorrhizal fungi (AMF) are ubiquitous soil microbes forming one of the most widespread and ancient symbioses on Earth, associating with the roots of *c*. 70% of land plants (Brundrett & Tedersoo, 2018). In this mutualistic relationship, plants exchange photosynthetically fixed carbon for essential nutrients, such as phosphorus and nitrogen, which AMF forage from the soil. AMF predominantly exist in the form of hyphae, which are the thread‐like, tubular structures that constitute the basic units of growth of all filamentous fungi. Collectively, a network of hyphal filaments is termed mycelium–a dynamic and adaptive structure responsible for soil exploration, nutrient foraging, host colonization, and environmental interaction. AMF mycelia play a crucial role in shaping soil structure and ecosystem dynamics by binding soil particles into stable aggregates, enhancing porosity, water infiltration, and aeration (Leifheit *et al*., 2014). These structural changes create microhabitats that support diverse microbial communities and reinforce ecosystem resilience (Powell & Rillig, 2018; Jia *et al*., 2021). At a broader scale, AMF mycelia can connect to multiple plants simultaneously, forming potentially indefinitely large underground common mycorrhizal networks (Giovannetti *et al*., 2004) that exert context‐dependent effects, ranging from positive to negative, on both plant and fungal traits (Lehmann *et al*., 2025).

The AMF mycelium bifurcates functionally and spatially: Intraradical mycelium (IRM) develops within root epidermal, subepidermal, and cortical cells, forming characteristic highly branched hyphal structures known as arbuscules that facilitate nutrient exchange with the host plant. Simultaneously, extraradical mycelium (syn. extramatrical) (ERM) proliferates into the soil matrix, extending the fungal reach far beyond the root zone to scavenge for nutrients and water. ERM and IRM consist of a single interconnected cell coined as coenocytic, where the cellular content is potentially shared across the entirety of the cell, till the moment in which septa are formed, separating the functioning hyphal segment from the senescing empty one (Cargill *et al*., 2025) (Supporting Information Video S1). AMF mycelia can be found growing across all soil types (Stürmer *et al*., 2018), colonizing roots of terrestrial (Brundrett & Tedersoo, 2018) and aquatic plants (Søndergaard & Laegaard, 1977), and even reaching the soil surface to colonize leaf litter (Bunn *et al*., 2019). Globally, it is estimated that up to 3.93 gigatons of CO_2_ annually are allocated to the ERM in soil alone, playing a potentially critical role in global carbon cycling and climate regulation (Hawkins *et al*., 2023). Examining how these extensive networks assemble, maintain, and remodel themselves is key to understanding the function and stability of these fungal networks in a changing world.

Historically, AMF mycelia have been studied as disconnected parts, with focus placed intraradically on the arbuscular nutrient exchange interface, leaving much of the mycelial biology fragmented across studies and taxa. Here, I aim to consolidate current knowledge of mycelial dynamics in AMF by exploring five interconnected themes: (1) the development and structural complexity of AMF mycelia, (2) the dynamic allocation strategies between intra‐ and extraradical components, (3) resilience mechanisms against disturbance and stress, (4) physical and ecological interactions among coexisting mycelial networks, and (5) the internal coordination of cellular contents across their coenocytic structure. In doing so, I aim to contextualize the mycelium not only as a developmental and ecological unit but also as a frontier for future exploration in AMF research.

## II. Morphology and morphogenesis

**Fig. 1:**
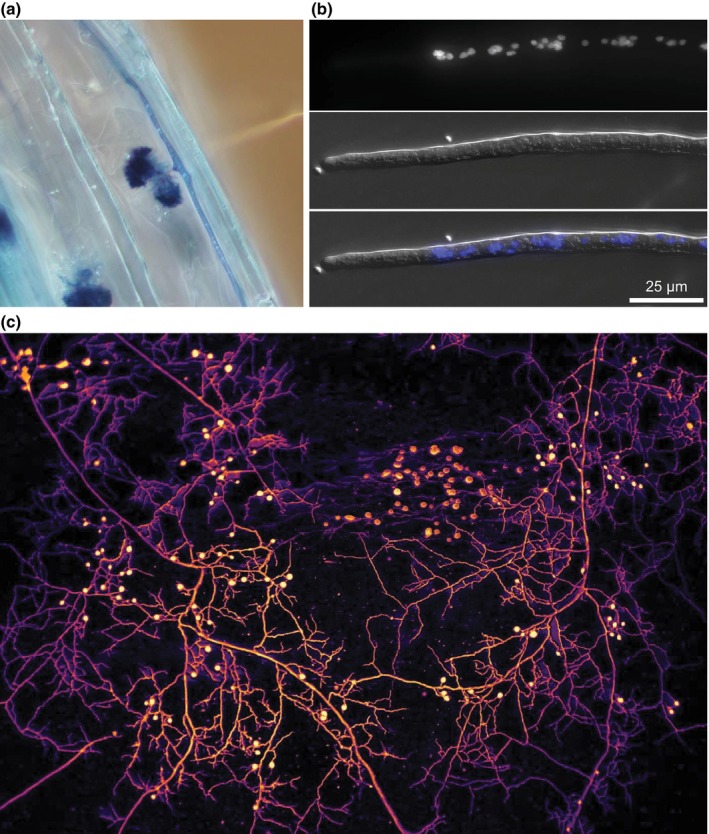
Conserved structures produced by the arbuscular mycorrhizal (AM) fungal mycelium. (a) Intraradical hyphae and arbuscules of the AM fungal species *Rhizophagus irregularis* stained with Trypan blue growing in the roots of *Linum usitatissimum*. Phosphorus delivery to the host takes place in arbuscules and is mediated by polyphosphate mobilization processes and phosphate (Pi) delivery to periarbuscular membrane‐localized proteins, such as PT4 (Luginbuehl & Oldroyd, 2017). In parallel, nitrogen transfer involves the breakdown of arginine within the fungus and the subsequent release of ammonium, which is taken up by periarbuscular membrane AMT channels on the plant side (Govindarajulu *et al*., 2005). (b) Multinuclear content and coenocytic cellular organization in a fixed germ tube of the AMF species *R. irregularis* DAOM197198. During germ tube growth, genes promoting fatty acid catabolism (e.g. FAL6 and FAL7) are upregulated, whereas genes related to nutrient uptake are strongly repressed, indicating that the germ tube prioritizes hyphal expansion over nutrient acquisition (Kameoka *et al*., 2019). Nuclei were stained with 0.5 μg ml^−1^ DAPI (4,6‐diamidino‐2‐phenylindole) for 10 min. Scale, 25 μm. (c) Extraradical mycelium consisting of runner hyphae, secondary and tertiary hyphal branches, branched absorbing structures (BAS), and spores, each serving a specific function. Genes encoding nitrate transporter, nitrate reductase, nitrite reductase, and trehalase are upregulated in runner hyphae, showcasing the active metabolism of this structure and its role in absorption and reduction of nitrate. BAS are linked to Pi absorption, showcased by the upregulation of PT1 and PT2 genes, which encode for H^+^/Pi symporters. In spores, genes encoding for aquaporins (AQP1) and for fatty acid modification (fatty acid elongase and delta 9‐fatty acid desaturase) reveal the importance of water transport to spore maturation and the role of spores in storing carbon (Kameoka *et al*., 2019). Artificial coloration was added for depth recognition, with brighter colours being closer to the observer. Image (b) is courtesy of Sander van Otterdijk. Image (c) is courtesy of Dr Loreto Oyarte Galvez.

**Fig. 2:**
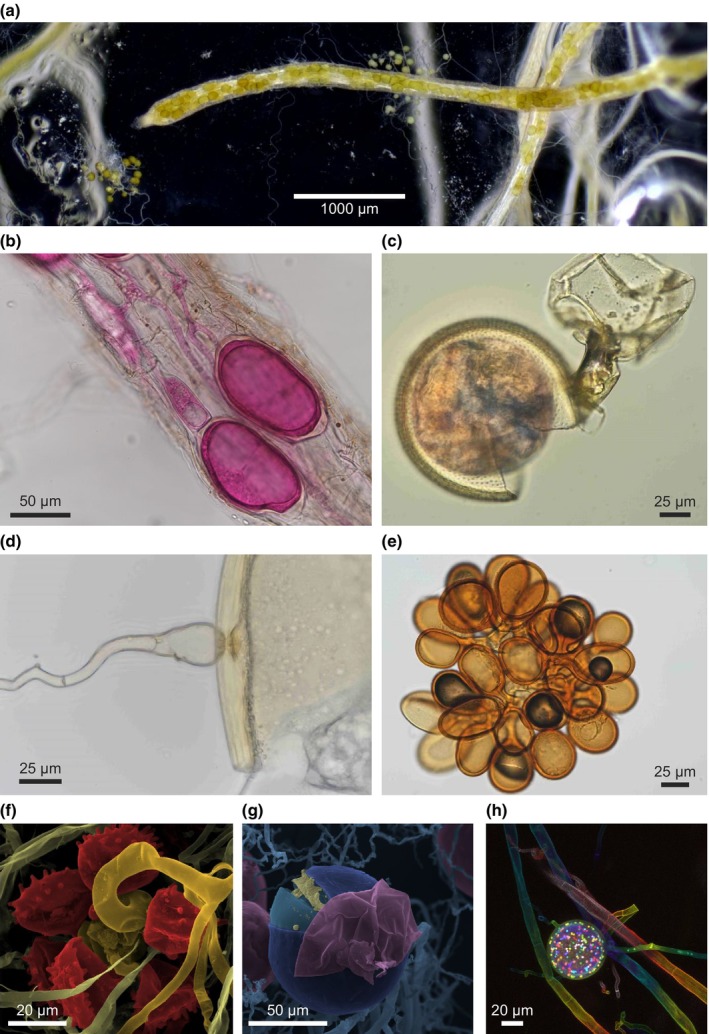
Phylogenetically distinct structures produced by the arbuscular mycorrhizal (AM) fungal mycelium. (a) *Rhizophagus irregularis* DAOM197198 vesicles growing densely within possibly metabolically inactive root tips of the host species *Plantago lanceolata* in the SAP‐based system (Paré *et al*., 2022). (b) *R. irregularis* vesicles and IRM growing in *P. lanceolata* roots stained with acid fuchsin. (c) Sporiferous saccule formed right behind an *Acaulospora scrobiculata* spore. The function of this structure is unknown. (d) Bulbous suspensor formed at the base of a *Gigaspora rosea* spore. (e) Sporocarp produced by *Sclerocystis* sp. in glasshouse trap culture conditions. (f) *Gi. margarita* auxiliary cells and extraradical hyphae seen via scanning electron microscopy and artificially colourised for structure differentiation. The function of this structure also remains unknown. (g) *R. irregularis* spores seen via scanning electron microscopy and artificially colourised to demonstrate the variable spore wall layers. (h) Multinucleate nature of *R. irregularis* DAOM197198 hyphae and spores with retention septa visible, distinguishing empty from active hyphae. Nuclei were stained with SYTO‐13 and visualized with confocal microscopy. Artificial coloration was applied for depth recognition. Image‐specific scales are placed directly in each item. Image (a) is courtesy of Louis Paré. Images (b–e) courtesy of Claudia Banchini, Canadian Collection of Arbuscular Mycorrhizal Fungi (CCAMF), Agriculture and Agri‐Food Canada (AAFC). © His Majesty the King in Right of Canada, as represented by the Minister of Agriculture and Agri‐Food (2020).

Though often portrayed as simple tubular filaments, AMF mycelia display remarkable functional complexity (Kameoka *et al*., 2019), characterized by distinct cellular organization (Bago *et al*., 1998a; Cargill *et al*., 2025) and specialized structure formation in both the extraradical and intraradical domains. Core architectural features include germ tubes, hyphopodia, runner hyphae, branched absorbing structures (BAS), and arbuscules, which are broadly conserved across all described AMF lineages (Fig. 1). By contrast, other structures such as spores, sporiferous saccules, bolbous suspensors, vesicles, auxiliary cells, and sporocarps show greater phylogenetic or ecological variability, appearing only in certain lineages or under specific environmental conditions (Fig. 2). The latter structures, despite being interconnected to the large coenocytic hyphal network and sharing protoplasmic continuity during development, are not considered part of the mycelium in strict mycological terms. Instead, together with the AMF mycelium, they comprise an integrated AMF network.

### 1. Germ tubes

The germ tube, the initial hyphal structure emerging from a spore, precedes the mycelium and transitions into differentiated hyphal growth during presymbiotic development, although a clear definition separating these phases remains lacking. In septate fungi, the transition from germ tube to hyphae is typically defined by elongation through mitotic growth followed by septum formation, which separates the original germ tube from the developing somatic hyphae (Sudbery, 2001). This definition does not readily apply to the coenocytic, multinucleate AMF. Branch initiation could alternatively mark the transition from germ tube to differentiated hyphae, a definition that fine‐scale spatial transcriptomics may help refine in the future. Germ tube emergence (germination) varies across phylogeny and appears to have three distinct mechanisms (Siqueira *et al*., 1985). (1) Germination from subtending hyphae, which is the hyphae that remains attached to spores post‐dispersal, taking place mainly in Glomeraceae, (2) direct germination from inner or outer spore wall layers as seen in the majority of AMF (Sward, 1981), and (3) emergence from peripheral compartments formed within spores, only seen in some Acaulosporaceae and Gigasporaceae taxa (Mosse, 1970; Hall, 1977).

At the cellular level, the germination process is understudied, with the majority of work on this topic taking place in the last century. Intriguing cellular occurrences happen during germination, pointing to the existence of tightly regulated cellular dynamics. These include the formation of structures known as germ pores in the genus *Gigaspora* (Khade, 2011) and the compartmentalization of spore protoplasmic space before germ tube emergence (Mosse, 1970). Such occurrences resemble the recently described cellularization processes in chytrids, which, following internal reorganization, transition from a multinucleate state to uniform uninucleate daughter cells (Medina *et al*., 2025). It is possible that compartmentalization during germination in AMF spores functions as a volumetric shorting mechanism, isolating a specific number of nuclei and cytoplasmic content. This function could be linked to controlled resource allocation, ensuring multiple germination events from a single spore to increase chances of survival (Koske, 1981). Previously established, as well as recently developed, protocols tailored to study nuclear, lipid, and other organelle dynamics in AMF (Cargill *et al*., 2025) should enable spatiotemporal cytological studies to better understand factors affecting the emergence and development of germ tubes.

Morphologically, germ tubes are thought to share a conserved architecture across AMF species: They are typically cylindrical and aseptate and expand by using the stored resources in a spore (Bago *et al*., 1998c; Maia & Yano‐Melo, 2001; Kokkoris *et al*., 2019b). However, comparative observations suggest subtle variations between species in the same genus, such as those in the *Gigaspora* genus, where germ tubes are shaped distinctly, with some being curved (*Gigaspora margarita*), and others straight (*Gi. decipiensor*) or coiled (unknown taxon) (Khade, 2011). Even larger differences can be seen between genera, with germ tubes of some Gigasporaceae species displaying negative geotropic growth (Becard & Fortin, 1988; Chabaud *et al*., 2002), a behaviour that is lacking in other families, or a differential response in terms of germination or branching intensity to plant‐derived chemical signals such as strigolactones (Besserer *et al*., 2006; Klein *et al*., 2024). These differences have not been functionally resolved but could point to diverse soil exploration and exploitation strategies that can influence the ability of germ tubes to locate and connect to plant hosts or existing hyphal networks (Cristiana Sbrana *et al*., 2011). Notably, the extent of germ tube and presymbiotic hyphal growth correlates with symbiotic mycelial development and could offer a rapid means to assess strain‐specific trait variation without the need to establish symbiotic cultures (Kokkoris *et al*., 2019b; Kokkoris & Hart, 2019b). Overall, understanding the physicochemical properties of AMF germ tubes could shed light on functional and phylogenetic variation across taxa while also enabling key methodological advances (Tominaga & Kaminaka, 2024). In particular, germ tubes may provide an ideal target for molecular techniques such as DNA/RNA and single‐molecule RNA fluorescence *in situ* hybridization (smFISH), cytological assays, and genetic transformation, due to their simpler structure and increased amenability to protoplast formation (Cargill *et al*., 2025; Woodward *et al*., 2025).

### 2. Hyphopodium

The hyphopodium, formerly referred to as appressorium, is a key morphogenetic structure marking the transition from germ tube, or differentiated presymbiotic hyphae, to root colonization. It also acts as a means for the ERM to re‐enter roots and further expand colonization or extend colonization to new plant hosts (Kiers *et al*., 2011). Structurally, it forms as a swollen, dome‐shaped interface hyphae accompanied by tip growth arrest (Genre *et al*., 2005) on the root epidermis, preparing the fungus for controlled entry into the epidermis and cortical root layers (Smith & Read, 2008). Unlike pathogenic fungi that develop high turgor pressures in their appressoria (up to 80 bars, equivalent to over 1100 pounds per square inch (psi)) to mechanically breach host surfaces (Howard & Valent, 1996), AMF rely primarily on enzymatic activity (Kinden & Brown, 1975; Scannerini & Bonfante‐Fasolo, 1983) and on sophisticated host cellular alterations to establish root entry, and as such, the terminology of the structure has been recently adjusted to avoid confusion. This process involves the assembly of the pre‐penetration apparatus (PPA), a coordinated reorganization of the plant's cytoskeleton and membranes that guides fungal ingress via the formation of cytoplasmic bridges, avoiding cellular rupture (Genre *et al*., 2005, 2008). The role of turgor pressure in this process, while of interest, remains unknown since no direct measurements of hyphopodia or hyphal turgor pressure in AMF have been reported to date. The formation of the hyphopodium appears to be strictly under fungal control and not rely on plant signaling since this structure can still be formed on top of extracted and purified plant epidermis cell walls (Nagahashi & Douds Jr, 1997). Notably, extracted epidermis of non‐host plants does not result in hyphopodium formation, pointing to the presence of a fungal‐based recognition mechanism able to discriminate between host and non‐host plant epidermal structures (Nagahashi & Douds Jr, 1997).

Although hyphopodium morphology appears conserved across AMF taxa (Tawaraya *et al*., 2007; Genre *et al*., 2008; Pepe *et al*., 2020), formation timing and frequency are AMF taxon‐specific (Pepe *et al*., 2020). Some taxa produce hyphopodia within 36 h (e.g. *Funneliformis mosseae*), while others can take up to 60 h (Giovannetti & Citernesi, 1993). Root age can also affect hyphopodia formation and frequency (Tawaraya *et al*., 2007), with younger roots typically showing higher susceptibility to colonization. This is not surprising since important alterations occur as the root matures, including loss of epidermal tissue (Wells & Eissenstat, 2002), which, as mentioned above, is crucial for host recognition and hyphopodia formation. Importantly, hyphopodia density is closely linked to phosphorus fluxes between soil and plant, highlighting that, beyond the IRM and ERM architecture and extent, the abundance and distribution of entry points to the root hold an important, but underexplored, role in resource exchange between the symbionts (Pepe *et al*., 2020).

### 3. Extraradical mycelium

**Fig. 3:**
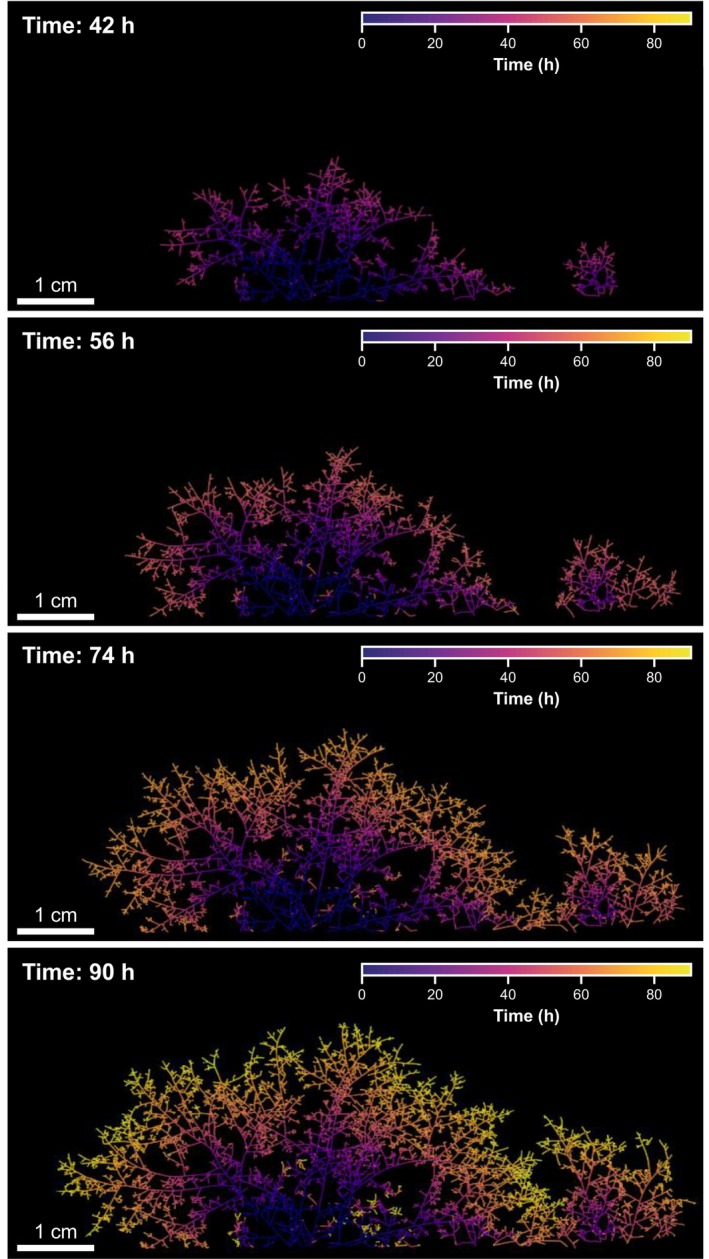
Extraradical mycelium (ERM) growth over time. *In vitro* ERM growth of the species *Rhizophagus irregularis* in symbiosis with *Daucus carota* root organ culture in a dual‐compartment Petri plate. The ERM growth is monitored in a fungal‐only compartment with a high‐throughput imaging robot (Oyarte Galvez *et al*., 2025). ERM expansion arises from self‐organized, wave‐like dynamics. Artificial coloration is linked to temporal growth ranging from 0 h (blue) to 90 h (yellow). Bars, 1 cm. Image courtesy of Dr Corentin Bisot.

ERM emerges rapidly following successful root colonization, but soil opacity makes it difficult to study under natural conditions. Despite its artificial nature, the establishment of *in vitro* root organ cultures (ROC) has enabled us to directly observe ERM architecture and growth dynamics (Becard & Fortin, 1988). Detailed investigations of the ERM of *Rhizophagus irregularis* and *Gigaspora rosea* reveal a modular architecture composed of coarse runner hyphae, secondary and tertiary branching zones, and finer BAS (Bago *et al*., 1998a,b,c). Runner hyphae facilitate long‐distance substrate exploration and support rapid, bidirectional cytoplasmic streaming for efficient nutrient and signal translocation across the network. BAS are densely branched and spatially associated with nutrient uptake zones and sites of sporulation, particularly where spore‐associated BAS develop (Bago *et al*., 1998c). Growth‐wise, the ERM follows a subapical growth pattern, where extension does not occur at the apex but instead behind the tip, enabling a sigmoid growth trajectory over time (Bago *et al*., 1998b; Silvani *et al*., 2014). Collectively, the hyphal network expansion is not the random cumulative effect of individual hyphal elongation, but it emerges from self‐organized, wave‐like dynamics (Oyarte Galvez *et al*., 2025). Our recent time‐lapse imaging and computational modelling revealed that AMF expand using synchronized pulses of hyphal tips, forming traveling waves that allow efficient substrate exploration while regulating local hyphal density (Fig. 3). Behind the advancing wavefront, hyphal self‐fusion events (anastomoses) strengthen network connectivity, optimizing nutrient transport back to the host and balancing exploration with internal coherence (Oyarte Galvez *et al*., 2025).

The densification of the network via hyphal self‐fusions is not consistent across all AMF lineages, reflecting distinct phylogenetically conserved strategies and ecological adaptations. Species within Glomeraceae, such as *Rhizophagus irregularis* and *Funneliformis mosseae*, are known to form dense, highly branched ERM with extensive hyphal interconnections in the presymbiotic and symbiotic mycelia (Giovannetti *et al*., 1999, 2001; de la Providencia *et al*., 2005; Oyarte Galvez *et al*., 2025). Fusion can even occur between genetically identical but distinct mycelia which originate from independent propagules and/or proliferate in different hosts (Giovannetti *et al*., 1999; Mikkelsen *et al*., 2008). Similarly, strains belonging to *Acaulospora* (*A. scrobiculata*, *A. spinosa*) and *Entrophospora etunicata* (syn. *Claroideoglomus etunicatum*) are also able to self‐fuse at high frequencies (Barreto de Novais *et al*., 2013). By contrast, members of the Gigasporaceae, including *Gigaspora margarita*, *Racocetra castanea* (syn. *Scutellospora castanea*), and *Scutellospora bireticulata*, produce thicker and longer hyphae with minimal or no self‐fusion events, which, when present, are in the form of hyphal bridges (de Souza & Declerck, 2003; de la Providencia *et al*., 2005) connecting different parts of the same hyphae (Giovannetti *et al*., 1999; de Souza & Declerck, 2003). These structural differences should be examined further as they might carry functional consequences: Dense, interconnected networks could provide greater nutrient exchange capability between symbiotic partners, greater resilience to disturbance, and could enable rapid resource redistribution. By contrast, sparse, non‐fusing taxa could rely more on reproductive output and on mycelial dispersal capacity by travelling longer distances and creating larger common mycorrhizal networks.

Beyond inherent growth patterns, ERM morphology is strikingly plastic, responding dynamically to physicochemical changes. Nutrient concentrations affect the density and architecture of ERM, and despite the continuous coenocytic nature of the network, these effects can take place locally, where segments of the network encounter heterogeneous conditions and adjust morphologically (Cornell *et al*., 2022). pH can have direct effects on the ERM, including alterations in hyphal density and enzymatic activity (van Aarle *et al*., 2002). Even physical obstacles can induce morphological changes by triggering hyphal branching and strategic biomass allocation to optimize environmental exploration and exploitation (Hammer *et al*., 2024). Furthermore, plant host identity (Giovannetti *et al*., 2004; Serghi *et al*., 2021; Cornell *et al*., 2022; Bisot *et al*., 2025) and plant host diversity (Engelmoer & Kiers, 2015) can also affect ERM, and in some cases, with negative consequences for the fungal partner, but little to no attention has been given to fungal benefit as a response to plant identity.

Historically, mycorrhizal response has been examined from a plant‐centric point of view in terms of partner fitness benefit. For example, the mycorrhizal mutualism–parasitism continuum is typically conceptualized from the perspective of the host plant, focusing on variations in plant benefit as a function of fungal identity (Johnson *et al*., 1997; Klironomos, 2003). However, this perspective overlooks the reciprocal dynamic of the symbiosis. From the fungal standpoint, host identity can exert a profound influence on fungal fitness, particularly in terms of ERM development and spore production (Serghi *et al*., 2021; Bisot *et al*., 2025). For example, hosts that are less reliant on AMF for nutrient acquisition (Serghi *et al*., 2021; Cornell *et al*., 2022) or exhibit reduced carbon allocation to their fungal partners (Bisot *et al*., 2025) can result in stunted or reduced ERM proliferation. In some cases, they can result in a net loss in fungal fitness, evidenced by the production of fewer daughter spores than the initial inoculum (Serghi *et al*., 2021; Cornell *et al*., 2022). Despite these findings, negative host effects on AMF development remain underexplored. This neglect stems from two persistent assumptions: First, that AMF, due to their generalist nature and broad host range, will invariably benefit from root colonization; second, that root colonization itself is synonymous with long‐term fungal success, due to the obligatory biotrophic lifestyle of these organisms. These assumptions conflate the presence of colonization with the quality of the symbiosis from the fungal perspective, highlighting the need for a better working definition of fungal fitness (Box 1). Shifting the focus to include fungal fitness responses challenges this paradigm and opens up new avenues for understanding the ecological consequences of host–fungal mismatches and host specificity or preference and their potential impacts on fungal population dynamics, community composition, and inoculum efficacy in managed ecosystems.

Box 1 **Why AMF fitness needs a clearer definition**

Defining fitness in filamentous fungi remains a challenging and often debated task (Pringle & Taylor, 2002; Gilchrist *et al*., 2006). In strict ecological terms, fitness represents the overall capacity of an individual to persist and produce offspring in a given environment, integrating survival, growth, and reproduction. However, the specific parameters that should be incorporated into a quantitative definition of fungal fitness remain uncertain, particularly for arbuscular mycorrhizal fungi (AMF), whose obligate asexual symbiotic lifestyle and coenocytic, multinucleate organization complicate straightforward application of conventional fitness metrics. In practice, AMF fitness is often inferred indirectly through measurable proxies such as daughter spore production, length of extraradical mycelium (ERM), and host root colonization. Each of these proxies reflects only a subset of the processes contributing to persistence and reproductive success, and each is influenced by environmental conditions, host identity, and methodological constraints.

For example, reproductive output is usually quantified as daughter spore production, ideally standardized per soil volume or mycelial biomass and time. Notably, AMF exhibit a spore size–number trade‐off where large‐spored taxa typically produce fewer but larger spores, implying allocation constraints relevant to fitness comparisons across species. Furthermore, many daughter spores are not viable, or they possibly do not serve as propagules (see Section IV), meaning that spore counts alone, without corresponding assessments of viability or infectivity, provide an incomplete and potentially misleading estimate of fitness. Other commonly used measures, such as ERM length from destructive soil extraction or intraradical mycelium (IRM) estimated by percentage root colonization, are also limited. These approaches do not capture the persistence of functional mycelium, its capacity to colonize neighbouring hosts, or its ability to regrow and reconnect following disturbance, despite the fact that these traits likely contribute substantially to lifetime reproductive output. Finally, successful colonization itself does not necessarily equate to increased AMF fitness. For instance, in a monoculture of a facultatively mycorrhizal plant that strictly regulates carbon allocation to its fungal partner, AMF propagule production may decline over time. While such associations may sustain short‐term survival of existing propagules (one generation), they can ultimately reduce reproductive capacity and risk local extinction of the AMF species across multiple generations.

It has been suggested that filamentous fungal fitness can be approximated by a single fitness metric such as spore number (Pringle & Taylor, 2002). Applying this approach to AMF, however, requires caution to ensure accuracy. For example, rather than relying solely on spore counts, the most probable number (MPN) method, which considers other propagule types in addition to spores (Porter, 1979), could provide a more comprehensive measure of reproductive output. Alternatively, fitness could be assessed through the expression of molecular markers associated with arbuscule formation and nutrient exchange (Duan *et al*., 2024), provided we develop a clearer understanding of how these markers relate to fungal survival, growth, and reproduction.

Ultimately, the challenge of defining AMF fitness highlights a deeper need to rethink conventional fitness concepts and calls for frameworks that integrate survival, reproduction, and persistence.

To fully understand ERM, we need to study more AMF taxa. Most studies remain limited to a narrow subset of taxa that can survive surface sterilization and can be cultured axenically (Becard & Fortin, 1988). Nonetheless, the prevailing assumption that only *Glomus* and *Rhizophagus* members are suitable for *in vitro* systems is not well‐founded. Multiple studies have demonstrated that a broad range of AMF taxa–spanning 10 genera–can be successfully cultured under *in vitro* conditions (Table 1). And even if axenic growth remained restrictive, the recently developed transparent soil system based on a superabsorbent polymer (SAP) (Paré *et al*., 2022) enables the growth of a broader range of AMF species in symbiosis with whole plants (Table 1). While this second system benefits from optical transparency, it still requires optimization for scalability and compatibility with high‐throughput microscopy (Oyarte Galvez *et al*., 2025). Nevertheless, it readily allows useful comparisons between ROC and natural hosts as well as sterile vs non‐sterile environments to help us understand how the artificial *in vitro* conditions might be influencing AMF (Kokkoris & Hart, 2019a). Future studies should focus on investigating ERM architecture and dynamics across phylogenetically diverse AMF in association with a wider range of ROCs, real host plant species, and across diverse environmental conditions.

**Table 1:** Arbuscular mycorrhizal (AM) fungal species successfully established either *in vitro* or in super absorbent polymer (SAP) cultures.

Genus	Species	Synonym	Spore sterilization	Host	Host type	Medium	Culture type	Citation
*Acaulospora*	*rehmii*		Yes	*Daucus carota*	ROC	MSR	*In vitro*	Dalpé & Declerck (2002)
*Diversispora*	*varaderana*		No	*Plantago lanceolata*	Seedling	mMS‐1/2	SAP	Paré *et al*. (2022)
*Entrophospora*	*lamellosa*		Yes	*Daucus carota*	ROC	MSR	*In vitro*	CCAMF^2^
*Funneliformis*	*mosseae*		Yes	*Linum usitatissimum*	ROC	MSR	*In vitro*	Rodrigues & Rodrigues (2015)
*Funneliformis*	*caledonius*		Yes	*Daucus carota*	ROC	M	*In vitro*	Karandashov *et al*. (2000)
*Funneliformis*	*geosporus*	*Glomus macrocarpum*	Yes	*Daucus carota*	ROC	MSR	*In vitro*	Declerck *et al*. (1998)
*Funneliformis*	*geosporus*	*Glomus macrocarpum*	No	*Plantago lanceolata*	Seedling	mMS‐1/2	SAP	Paré *et al*. (2022)
*Gigaspora*	*margarita*		Yes	*Daucus carota*	ROC	MSR	*In vitro*	de la Providencia *et al*. (2005)
*Gigaspora*	*rosea*		Yes	*Daucus carota*	ROC	MSR	*In vitro*	de la Providencia *et al*. (2005)
*Gigaspora*	*margarita*		Yes	*Lycopersicon esculentum*	ROC	MS	*In vitro*	Miller‐Wideman & Watrud (1984)
*Gigaspora*	*gigantea*		Yes	*Daucus carota*	ROC	M	*In vitro*	Gadkar *et al*. (1997)
*Gigaspora*	*rosea*		No	*Plantago lanceolata*	Seedling	mMS‐1/2	SAP	Paré *et al*. (2022)
*Glomus*	*versiforme*		Yes	*Lycopersicon esculentum Daucus carota*	ROC	M/MSR	*In vitro*	Diop *et al*. (1994); Declerck *et al*. (1996)
*Oehlia*	*diaphana*		Yes	*Daucus carota*	ROC	MSR	*In vitro*	CCAMF/GINCO^3^
*Racocetra*	*fulgida*		No	*Plantago lanceolata*	Seedling	mMS‐1/2	SAP	Paré *et al*. (2022)
*Rhizophagus*	*clarus*		Yes	*Daucus carota*	ROC	MSR	*In vitro*	CCAMF
*Rhizophagus*	*intraradices*	*Glomus intraradices*	Yes	*Daucus carota*	ROC	M	*In vitro*	Chabot *et al*. (1992)
*Rhizophagus*	*irregularis*		Yes	*Daucus carota*	ROC	M	*In vitro*	Bago *et al*. (1998b)
*Rhizophagus*	*fasciculatus* ^1^		Yes	*Daucus carota*	ROC	MSR	*In vitro*	Declerck *et al*. (1998)
*Rhizophagus*	*prolifer*	*Glomus proliferum*	Yes	*Daucus carota*	ROC	MSR	*In vitro*	Declerck *et al*. (2000)
*Rhizophagus*	*intraradices*	*Glomus intraradices*	No	*Plantago lanceolata*	Seedling	mMS‐1/2	SAP	Declerck *et al*. (1998); Paré *et al*. (2022)
*Sclerocystis*	*sinuosa*		Yes	*Daucus carota*	ROC	M	*In vitro*	Bi *et al*. (2004)
*Scutellospora*	*reticulata*		Yes	*Daucus carota*	ROC	MSR	*In vitro*	de la Providencia *et al*. (2005)
*Septoglomus*	*deserticola*		Yes	*Ziziphus nummularia*	Sterile plantlet	MS	*In vitro*	Mathur & Vyas (1995)

^1^ Possibly *R. irregularis*, see Kokkoris *et al*. (2024).

^2^ Canadian Collection of Arbuscular Mycorrhizal Fungi (CCAMF) – https://agriculture.canada.ca/en/science/collections/canadian‐collection‐arbuscular‐mycorrhizal‐fungi‐ccamf.

^3^ Glomeromycota *In vitro* Collection (GINCO) – https://www.mycorrhiza.be/ginco‐bel/catalogue.php.

Genera and species nomenclature were adjusted after http://www.amf‐phylogeny.com/.

### 4. Intraradical mycelium

While the ERM extends outward to forage for soil resources, IRM colonizes plant roots by growing between and within cells, forming runner hyphae and highly branched structures such as arbuscules or coils in cortical cells. Some AMF species are capable of producing additional structures known as vesicles, which, although commonly referred to as lipid storage units, are active propagules capable of germinating and initiating plant colonization following extraction from root tissue (Declerck *et al*., 1998). Overall, the stored carbon in the IRM can sustain fungal growth for extended periods even after host shoot removal (Pepe *et al*., 2018).

Arbuscules are possibly the most well‐characterized parts of the AMF mycelium and are key for nutrient exchange between partners. The arbuscule's intricate, tree‐like morphology maximizes surface area for metabolic exchange and arises through coordinated cytoskeletal reorganization, membrane remodelling, and complex signalling, processes that have been thoroughly reviewed and will not be detailed here (Pumplin & Harrison, 2009; Luginbuehl & Oldroyd, 2017; Ivanov *et al*., 2019). Recent advances in live cell fluorescence imaging, such as using fluorescent phosphate transporters in combination with innovative live‐imaging systems (Pumplin & Harrison, 2009; Kobae & Hata, 2010; McGaley *et al*., 2025), enabled monitoring IRM progression and arbuscule life cycle in great detail. These methods have shown that arbuscules are short‐lived, needing 1–2 d to develop, 1–4 d to fill the cell, and 1 d to collapse. Following collapse, arbuscule remnants diminish within 3–5 d (McGaley *et al*., 2025). But despite their transient nature, AMF can cycle through repeated arbuscule formation events within the same root system, sustaining long‐term colonization via a nomadic lifestyle, preferentially forming new arbuscules in previously unoccupied cells (Kobae & Fujiwara, 2014).

Historically, IRM has gained more attention because it is easier to visualize thanks to many staining and colonization quantification protocols (Trouvelot *et al*., 1986; McGonigle *et al*., 1990; Vierheilig *et al*., 1998). While these approaches are informative for the status and extent of mycorrhization, they are inconsistent when we attempt to link them to function (Smith & Smith, 2011; Kokkoris *et al*., 2019c), demonstrating a non‐linear, and other times, unpredictable relationship (Frew, 2025). An important factor contributing to this disparity is that trait variation present in IRM which links to function is ignored. IRM growth patterns span a morphological continuum known as the Arum–Paris‐type, with Arum‐type AM characterized by intercellular hyphae and arbuscules, and Paris‐type by intracellular coils and/or arbusculate coils, where arbuscules are formed intercalary of coils (Brundrett & Kendrick, 1988, p. 199; Cavagnaro *et al*., 2001). Expression of these morphotypes is influenced by both host and fungal identity as well as root age, with the same plant species forming different structures depending on the fungal partner and sometimes exhibiting both types within a single‐root system (Smith & Smith, 1997; Dickson *et al*., 1999; Cavagnaro *et al*., 2001). These diverse architectures have been linked to mycorrhizal response efficiency, with coils hypothesized to be a less mutualistic interface for both partners. Supporting this hypothesis, Kobae & Hata (2010) showed phosphate transporter activation in arbuscules but not in coils of either *F. mosseae* or *Gi. rosea*, showcasing that just looking for the presence or absence of the IRM and grouping arbuscular/coil abundance is not enough to demonstrate a successful symbiosis. Notably, expression of plant symbiotic Pi‐transporters (e.g. *Medicago* PT4) and regulators (phosphate starvation response modules) tracks nutrient delivery more closely than total colonization values, and loss of these pathways decouples mycorrhization from benefit (Breuillin‐Sessoms *et al*., 2015; Wang *et al*., 2024), indicating that plant gene activity could be an informative trait when evaluating symbiotic efficacy.

At a spatiotemporal scale, IRM growth in roots depends on multiple factors, including host species, cultivar identity, soil conditions, and AMF species or strain and can range from as little as 0% (failure to colonize) to as much as 100% within compatible hosts. For example, colonization timing varies among species and genera spanning from 1 to > 6 wk, reflecting important growth speed variability (phylogenetically preserved) (Hart & Reader, 2002) that can eventually affect the overall AMF community composition within roots (Gao *et al*., 2019). But how this succession takes place at the cellular level is not well described. Key questions include whether these slower colonizers can exploit pre‐formed intraradical mycelial networks or benefit from physiological and structural changes left behind by their predecessors. Whether they can successfully form new arbuscules in root cortical cells that previously hosted arbuscules from other species or whether cellular residues from previously collapsed arbuscules in the recolonized cells (e.g. Ca dispositions) could provide desirable resources to taxa that follow.

Host identity also strongly influences IRM development, affecting not only colonization frequency but also internal hyphal density (Hart & Reader, 2002; Koch *et al*., 2017). Hosts with high mycorrhizal dependency or low nutrient acquisition efficiency, such as legumes or grasses, or plants with coarse root systems (Hetrick *et al*., 1992) promote greater IRM proliferation and arbuscule development. Cultivar identity can likewise have a strong effect on the IRM establishment and growth. For example, IRM colonization of a single AMF strain (*Rhizophagus irregularis*) ranged from 21 to 89%, and 42% of the variation was explained by rice genotype after examining 334 rice cultivars (Davidson *et al*., 2019). Besides the host, IRM growth and dynamics are strongly influenced by abiotic conditions. High soil phosphorus and low light availability both suppress IRM development by limiting plant–fungal interaction and by reducing the carbon supplied from the host plant, respectively. While these abiotic conditions often affect both ERM and IRM similarly, others can elicit divergent responses; for example, IRM colonization may increase at lower temperatures while ERM development is inhibited (Tibbett & Cairney, 2007). These decoupled responses suggest that IRM and ERM function as a plastic, semi‐independent system capable of differential adaptation to environmental conditions despite their unicellular nature.

## III. Allometry (IRM : ERM)

Allometry refers to the differential growth of organismal parts relative to overall size, resulting in changes in proportions. The balance between ERM and IRM development can be conceptualized through an allometric framework, analogous to the root‐to‐shoot ratio in plants (Johnson *et al*., 2003). This ratio reflects the allocation of resources between soil exploration (ERM) and root colonization (IRM), influencing the efficiency of nutrient uptake, nutrient allocation, and nutrient exchange. For instance, some AMF strains exhibit extensive ERM networks with limited IRM development (edaphophilic), and others invest more in IRM while limiting ERM growth (rhizophilic). These allometric strategies are influenced by both fungal genetics and environmental factors, including host identity and soil nutrient availability.

A range of quantification approaches shows phylogenetically conserved traits but also substantial inter‐/intraspecific variation. For example, tracking AMF mycelia using ergosterol measurements shows that Glomeraceae species (*R. irregularis*, *F. mosseae*, *F. geosporus*) typically exhibit rapid and extensive root colonization with constraint close to the root ECM, suggesting a strategy prioritizing immediate access to host carbon and *in situ* nutrient exchange. By contrast, Gigasporaceae taxa (*Gi. gigantea*, *Gi. margarita*, *S. calospora*, *S. heterogama*, *S. pellucida*) display robust extraradical development with limited root colonization, pointing towards a strategy oriented around expansive soil foraging and possibly delayed symbiotic pay‐off. Some Acaulosporaceae species (*A. spinosa*, *A. morrowaie*) occupy an intermediate or restrained niche, often showing low biomass in both compartments (Hart & Reader, 2002) and in some cases being entirely absent in soils, existing only intraradically (Volpe *et al*., 2023), reflecting increased host dependency and conserved growth strategies. Other Acaulosporaceae species display opposing trends with *A. laevis* maintaining constant hyphal density able to extend > 10 cm from host roots (Jakobsen *et al*., 1992), demonstrating the large interspecific variation even between phylogenetically related species. Notably, metabarcoding datasets show that members of Archaeosporaceae and Paraglomeraceae have a strong presence in the soil but are limited (Větrovský *et al*., 2023) or entirely absent within nearby roots (Hempel *et al*., 2007), raising the possibility that certain AM lineages may exhibit strong host preference, reduced or transient IRM phases, or may even engage in alternative (other than plant) symbiotic interactions or facultatively biotrophic lifestyles.

Besides well‐established morphogenetic patterns, the ERM : IRM ratio remains plastic and appears to be governed, at least in part, by principles consistent with the functional equilibrium model (Johnson *et al*., 2003). This model posits that organisms optimize their biomass allocation towards structures that acquire the most limiting resource (Chapin *et al*., 1990). Applied to AMF, this means dynamically shifting investment between ERM and IRM depending on the relative availability of soil nutrients and host‐derived carbon. Abiotic factors such as soil nutrient levels (Johnson *et al*., 2003) and type of fertilization (mineral vs organic) (Gryndler *et al*., 2006) elicit such responses, with N enrichment reducing allocation to arbuscules in the IRM (Johnson *et al*., 2003) and mineral fertilization reducing overall IRM, and altering the ERM dynamics by enriching spore production at the expense of extraradical hyphae (Gryndler *et al*., 2006). Finally, host plant species identity and AMF species coexistence can drastically affect the ERM : IRM ratio. Different hosts can trigger varying degrees of ERM : IRM plasticity (Kokkoris *et al*., 2019a), which appears more extreme within (intraspecific variation) rather than between species (interspecific variation) (Koch *et al*., 2017). Also, competition among coexisting strains shifts the investment of the ERM : IRM ratio per strain when sharing a host (Engelmoer *et al*., 2014).

Yet, despite the significance of these findings, most should not be viewed as definitive; instead, as phylogenetic relationships and quantification techniques are refined (Table S1), we should aim to re‐investigate these dynamics at finer spatial and temporal scales. For example, ERM is usually quantified as total hyphal length (e.g. cm g^−1^ soil), while IRM is commonly quantified as % colonization, making direct comparisons with such methods unsuitable. Chitin and ergosterol‐based assays could be significantly skewed by the presence of other fungi and are not perfectly suitable for environmental samples (Varma, 2008). Quantification of phospholipid fatty acid (PLFA) 16 : 1ω5 is also unreliable for environmental samples due to the lack of signal specificity, which can also include bacteria PLFA 16 : 1ω5 (Varma, 2008). High‐throughput imaging and techniques that allow for temporal observations are designed exclusively for IRM (Kumar *et al*., 2022; McGaley *et al*., 2025) or ERM (Oyarte Galvez *et al*., 2025) but not for both simultaneously. Quantification of neutral lipid fatty acid (NLFA) 16 : 1ω5 can result in meaningful comparisons when, at least, the genus *Gigaspora* is not present, which can result in biomass misestimation due to its low content in NLFA 16 : 1ω5 (Varma, 2008).

Finally, DNA quantification methods could prove the most efficient, but first, they have to meet specific requirements that are usually neglected. Primarily, we have to design molecular tools appropriate for quantitative approaches (qPCR, ddPCR) targeting specific groups across phylogeny (across all AMF, at family level, etc.) to allow for comparative studies. While some tools already exist (Bodenhausen *et al*., 2021; Corona Ramírez *et al*., 2023), they present several limitations. First, correlating visual colonization estimates with molecular copy numbers can be misleading. Microscopy‐based assessments often include stained necromass which lacks nuclei and is undetectable by DNA‐based methods. Second, established methods using visual scoring typically overlook the actual intensity or density of hyphae within a given root segment, potentially leading to large discrepancies between morphological and molecular estimates of colonization (Kokkoris *et al*., 2019c). Furthermore, these tools rely on ribosomal DNA (rDNA) as the molecular target. rDNA is not a single‐copy gene. This complicates efforts to relate gene copy number to meaningful measures of physical abundance, such as hyphal length or biomass.

Additionally, considerable intragenomic variation exists among the rDNA copies within a single AMF genome (Maeda *et al*., 2020), which can introduce amplification bias during PCR, preferentially amplifying certain rDNA variants over others and skewing estimates of community composition or abundance. An alternative solution is to instead target single‐copy genes (e.g. MAT‐loci and tubulin) which physically relate to single nuclei linking copy number to physical meaning (Kokkoris *et al*., 2021). It has been shown before that nuclear distribution in the AMF mycelium is not random but instead nuclei appear at regular intervals (Bago *et al*., 1998a,c; Oyarte Galvez *et al*., 2025) (e.g. on average every 2.4 μm in the mycelia of *R. irregularis*), making it possible to link nuclear abundance to mycelial length. It becomes apparent that linking molecular tools to meaningful AMF quantification in environmental samples requires a better understanding of the cellular organization of the hyphae and in particular of the nuclear spatial distribution in the coenocytic AMF mycelia (Cargill *et al*., 2025).

Overall, AMF balance resource allocation between ERM and IRM, with strategies ranging from prioritizing extraradical investment to emphasizing root colonization. This variation is shaped by phylogenetic history, host identity, and environmental conditions, while plasticity in the ERM : IRM ratio reflects functional equilibrium principles. Together, these patterns highlight both conserved growth strategies and ecological flexibility and point to the need for improved, standardized methods to more accurately quantify fungal investment dynamics across taxa.

## IV. Robustness and resilience

In natural settings, AMF must constantly balance survival strategies against pressures from predation (Warnock *et al*., 1982; Jiang *et al*., 2020), mycoparasitism (De Jaeger *et al*., 2010), competition (see section ‘interacting mycelia’), and physicochemical disturbances (Hildebrandt *et al*., 2007; Schnoor *et al*., 2011; Ipsilantis *et al*., 2012) that could reduce their lifespan. The multilayered and robust AMF cell wall serves as the first line of defence. It is therefore surprising that the cell wall remains structurally unresolved (Cargill *et al*., 2025). Beyond understanding what it is made of and how it's organized, future work should also examine whether (and if yes, how) dynamic cell wall structural remodelling takes place in AMF in response to environmental stress, similarly to other fungal groups (Hopke *et al*., 2018; Fernando *et al*., 2023). Identifying signalling components responsible for such responses could be a useful tool in boosting AMF immunity under adverse conditions via exogenous application.

The resilience of AMF mycelia extends beyond their physical robustness to encompass impressive cellular adaptability. Although the ERM has been reported to exhibit rapid turnover under field conditions, surviving only 5–7 d (Atkinson & Watson, 2000; Staddon *et al*., 2003), this dynamic likely reflects the turnover of fine hyphal branches, such as BAS, which are known to empty and become septated approximately a week after their formation (Bago *et al*., 1998a), rather than wholesale protoplasmic loss. In fact, other carbon tracking experiments suggest that AMF retain assimilated carbon for at least 32 d, particularly when predation and microbial pressure are excluded, and up to many months when growing *in vitro* (Olsson & Johnson, 2005), indicating that much of the protoplasm remains viable even as older or finer hyphal compartments degenerate. This becomes possible due to the ability of AMF to dynamically relocate their protoplasm across the network, forming retention septa that isolate emptied hyphae, and in some cases, even reverse this process by repopulating abandoned hyphae through septum removal (Lee, 2011). Protoplasmic migration across the entirety of a network and protoplasmic longevity have never been methodologically examined. It is fascinating to think that the protoplasmic dynamics, if studied at the mycelium scale, could show similarities to plasmodial amoeboid locomotion (e.g. *Physarum* sp.) (Lewis *et al*., 2015), with the AMF protoplasm relocating freely within the cell wall boundaries of the ERM and IRM, potentially abandoning non‐efficient trade routes.

Unlike septate fungi, the coenocytic hyphae of AMF permit continuous cytoplasmic streaming, creating a fully interconnected cellular system. While this connectivity enhances nutrient translocation, it raises fundamental questions about how AMF buffer local damage or stress, given the absence of internal compartmentalization. Although coenocytic, AMF can form retention septa (Fig. 2h) to regulate arbuscule lifespan (Kinden & Brown, 1975; Scannerini & Bonfante‐Fasolo, 1983) and to rapidly compartmentalize injured or senescing hyphal segments. This is crucial for preserving network integrity and enabling regeneration and network reconnection through renewed hyphal growth which occurs in < 24 h (de la Providencia *et al*., 2005). We do not know whether all AMF species share this ability, but we do know that phylogenetically distinct strategies exist. *Glomeraceae* members initiate hyphal regrowth through the septa, generating multiple hyphal tips that anastomose with different hyphae, while members of *Gigasporaceae* produce new hyphal tips behind the septa from both sides of a cut and orient to meet and reconnect the same hyphae via a hyphal bridge (de Souza & Declerck, 2003; de la Providencia *et al*., 2005; Rodriguez‐Morelos *et al*., 2021).

Another hypothetical resilience‐related mechanism is protoplasmic leakage prevention following hyphal disturbance. After physical damage to a hypha, it takes up to 15 min for a septum to be formed (de la Providencia *et al*., 2005), meaning that during this time protoplasm should be continuously leaking, especially considering the expected pressure differences between the intra‐ and extracellular space (turgor pressure). Instead, protoplasmic leaks, although present (de Souza & Declerck, 2003; de la Providencia *et al*., 2005), do not last more than a few seconds, after which a clotting mechanism is rapidly enabled. The exact mechanism responsible for this response is unknown in AMF, but similar mechanisms have been described in other fungal groups. In septate fungi, special organelles known as Woronin bodies are partially responsible for sealing septal pores in response to damage, preventing protoplasmic loss (Markham & Collinge, 1987; Steinberg *et al*., 2017; Mamun *et al*., 2023). Woronin bodies or Woronin‐like structures have never been observed in AMF mycelia, despite extensive transmission electron microscopy (TEM)‐based investigations; instead, a system similar to what was recently described in the closely related early‐diverging Mucorales (phylum Mucoromycota) could take place in AMF. Mucorales have a group of Gellin proteins with diverse properties that, when activated during disturbance, result in protoplasmic gelation (Nguyen *et al*., 2021). Although these specific Gellins were not detected in Glomeromycotina (*in silico*), this does not exclude the possibility that other analogous systems may exist and perform similar functions within AMF mycelia (Nguyen *et al*., 2021). For example, proteins associated with septation and septal pore plugging in *Aspergillus oryzae* (SPPA/C/D/J/P/B) have identifiable orthologs in AMF (Mamun *et al*., 2023), suggesting they may play important roles in septum formation and disassembly during healing or empty hyphae recolonization and thus warrant further investigation.

While Gellin‐like and SPP‐like proteins might protect AMF from physical disturbances, other proteins could be responsible for mediating other abiotic stressors such as sub‐zero temperatures. Soil temperatures as low as −3.3°C induce hyphal dormancy and extend ERM survival, enabling it to overcome seasonal freezing without losing infective capacity (Addy *et al*., 1994, 1997, 1998). This strategy appears to be linked to specific cellular alterations where the protoplasm is maintained in discontinued parts of the hyphae that are not connected to the host and are isolated by septa. These hyphal fragments can initiate new infective growth when favourable conditions return, indicating that new colonization can also occur solely from hyphae. It is intriguing to think that AMF species that appear only in eDNA analysis (Lutz *et al*., 2025) and do not have a described spore identity might propagate solely via hyphae rather than spores. The exact molecular mechanism responsible for protection from frost remains elusive. It is possible that AMF could possess antifreeze proteins exhibiting thermal hysteresis activities similar to the ones seen in psychrophilic fungi (Xiao *et al*., 2010; Das *et al*., 2022).

Mechanisms for tolerating extreme temperatures and drought must likewise operate in AMF. In other fungal systems, trehalose (a nonreducing disaccharide of glucose) can stabilize proteins and preserve membrane integrity during heat (Hottiger *et al*., 1987) and desiccation (Tapia & Koshland, 2014; Tapia *et al*., 2015). Its protective action is linked to the regulation of intracellular diffusion through modulation of cytoplasmic viscosity, a process coined ‘viscoadaptation’ (Persson *et al*., 2020). By increasing cytoplasmic viscosity, trehalose restricts molecular mobility, helping maintain homeostasis under stressful conditions. Upregulation of trehalose has been observed in AMF (*R. irregularis*) during heat shock and chemically induced stress (Ocón *et al*., 2007), which then returns to normal levels after stress has ceased. Future research should investigate the precise mechanisms of trehalose‐mediated protection in AMF, including their capacity for recovery after transient stress and the potential role of exogenously applied trehalose in enhancing resilience to environmental challenges such as in agricultural settings.

Protection from toxins is also important for soil fungi. AMF, especially in agricultural settings, are exposed to many toxic substances (e.g. heavy metals, pesticides, herbicides, fungicides, high nutrient content). The fact that AMF are present in these soils suggests that they might possess sophisticated detoxification mechanisms. Although some taxa are more tolerant than others for particular stressors (Lenoir *et al*., 2016), with local adaptation playing an important role in this (Rúa *et al*., 2016), most studies have adopted a plant‐centric view on this topic (Khalvati *et al*., 2010; Wu *et al*., 2018; Zhang *et al*., 2021), and fungal mechanisms remain largely unknown. While some information can be derived from *in silico* analysis (see Ferrol *et al*., 2016 and references within), how detoxification operates at the mycelial level is unknown. To my knowledge, only one study has directly demonstrated controlled spatial compartmentalization of excess toxins (in this case Cu) in a subset of second‐generation spores in *Rhizophagus irregularis*. These spores turn blue‐green due to Cu overaccumulation and are metabolically inactive (Cornejo *et al*., 2013). Indirect evidence also exists. It has been shown that under high mineral fertilization, field spore production may increase, but a significant portion of the resulting spores are dead. These findings point towards an understudied strategy where some spores might have diversified functions, acting as ‘garbage bins’ instead of propagules, facilitating localized detoxification for next spore generations. This warrants further exploration to understand how and which spores are chosen for distinct functions, as well as what fitness consequences such a mechanism might bring. I would expect a short‐term decline in fungal fitness due to the accumulation of toxins in spores, potentially rendering them non‐viable. However, this effect is likely to diminish over time as subsequent spore generations emerge in a detoxified environment. It is also important to highlight that the fact that spores can change colour so drastically based on the accumulated toxins needs to be accounted for when identifying environmental samples and assessing spore traits (Antunes *et al*., 2025; Chaudhary *et al*., 2025). Future studies should also explore bottom‐up trophic interactions by considering organisms that prey on detoxifying spore structures of AMF, as these interactions may influence broader soil food web dynamics.

Concluding, AMF appear able to endure diverse biotic and abiotic pressures through deterministically controlled cellular dynamics, stress‐response mechanisms, and potential detoxification strategies. Such a multi‐resilience approach likely contributes to their persistence across time and across ecosystems, but its mechanistic foundations require deeper study.

## V. Interacting mycelia

Roots are commonly colonized by multiple AMF taxa simultaneously. Studies using amplicon sequencing have reported anywhere from three to over 35 virtual taxa coexisting within individual, naturally occurring root systems (Clapp *et al*., 1995; Opik *et al*., 2008). Such multi‐taxon colonization creates opportunities for both synergistic and antagonistic interactions, acting both on plants and AMF, within a common host or the shared soil matrix.

For example, increased AMF richness does not consistently translate into increased host benefit: While some hosts seem to benefit from high AMF diversity in their roots (van der Heijden *et al*., 1998), fungal functional redundancy or antagonism can reduce overall symbiotic efficiency (Thonar *et al*., 2014). A suggested mechanism for predicting co‐inoculation outcomes has its basis on trait‐based approaches and how functional traits of coexisting organisms (1) fungal‐fungal and (2) plant‐fungal, are subjected to functional complementarity (Koide, 2000; Smith *et al*., 2000). In the first case, fungal species with different but complementary and phylogenetically conserved life history strategies have been seen to increase symbiotic efficiency when colonizing the same host (Smith *et al*., 2000) and to sustain long‐term higher AMF diversity in roots (Maherali *et al*., 2007). The same can occur when plant traits complement those of specific AMF taxa, creating functionally efficient mycorrhizal combinations (Koide, 2000; Chagnon *et al*., 2013). However, expectations based solely on complementary life history traits can oversimplify the complex dynamics involved. For example, despite complementary ERM and IRM dynamics, co‐inoculations with *Gigaspora* and *Rhizophagus* species can result in negative mycorrhizal responses in terms of biomass (Thonar *et al*., 2014). Examples like this emphasize the complexity of mycorrhizal interactions which are shaped by numerous functional traits and context‐dependent ecological variables. This further highlights the need for more comprehensive trait‐based frameworks that move beyond the limited commonly used mycelial traits (ERM abundance, arbuscular, vesicular, hyphal, and overall colonization) (Aguilar‐Trigueros *et al*., 2015; Antunes *et al*., 2025) to also consider the dynamic temporal nature of the symbiosis and the succession in interactions (Box 2).

Box 2 **Time‐dependent mycelial carbon requirements and their potential role in mycorrhizal response**

Despite extensive efforts, predicting plant response to arbuscular mycorrhizal fungi (AMF) remains challenging, possibly because studies often overlook the plant carbon (C) supply–demand dynamics and its link to AMF life cycle, plant ontogeny, and abiotic factors. Here, I briefly propose a simple supply–demand framework that centres on the temporal availability of plant‐derived C and the C demand of a growing mycelium:

C supply to the roots, and therefore partially to AMF, is determined by two trajectories:

1. A fixed allometric trajectory governed by plant developmental stage (early growth, flowering, seed/fruit production, and senescence) and life history (e.g. annuals, perennials, tubers). In this example, corn, an annual plant, is expected to allocate less C towards the roots over time (green solid line) by keeping increasingly more C aboveground to support flower and seed production.
2. A flexible trajectory shaped by environmental factors, such as nutrients, light, and water availability, which can change the root:shoot investment, shifting C dynamics. For example, increased C allocation to roots can be the result of low soil phosphorus, low nitrogen, and limited water. Decreased C allocation to roots can happen during light‐limited conditions, shifting the fixed trajectory upwards or downwards, respectively (dotted lines).

Meanwhile, AMF C demand (blue line) is related to mycelial development, which starts with low C demands during the initial colonization stages and increases steeply during spore/vesicle formation. Eventually, propagule dormancy and hyphal senescence are induced, reducing C fungal demands.

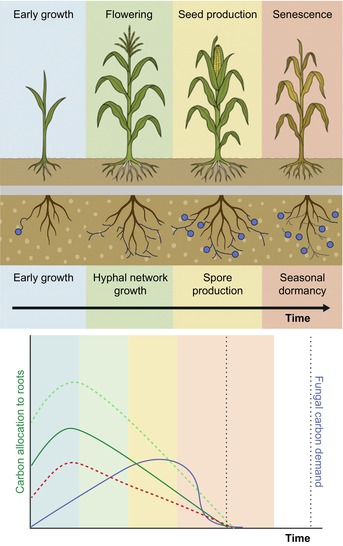

When the available plant C supply meets or exceeds fungal demand, the interaction could likely be mutualistic (mycorrhizal response > 1); when supply falls short, it may turn parasitic (mycorrhizal response < 1). Plants with consistent belowground C investment, such as tuberous species, are expected to show the most positive arbuscular mycorrhizal (AM) responses, while annuals with reduced C root allocation over time may benefit less, especially as mycorrhizal responses are examined in later plant life stages. A step further would involve including multiple AMF partners and their succession in the root to better understand diversity dynamics and under which conditions higher diversity could lead to beneficial or parasitic interactions. This conceptual model suggests that plant ontogeny, AMF mycelial temporal dynamics, and environmental conditions could help explain a considerable portion of AM symbiotic outcomes.

**Fig. 4:**
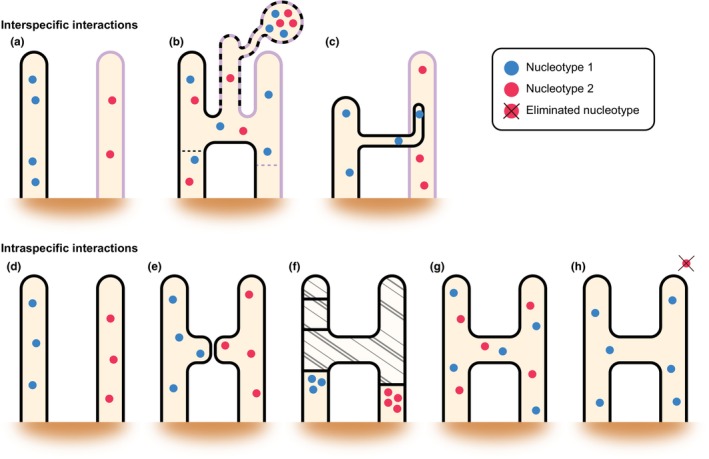
Interspecific (upper panel) and intraspecific (lower panel) arbuscular mycorrhizal (AM) fungal mycelial interactions. Upper panel: (a) Non‐interacting, where hyphae grow past one another without interaction. (b) Introgression, a hypothetical interaction where perfect fusion might occur between species, especially under increased stress/disturbance, resulting in hybrid non‐viable spores of intermediate morphology (dotted line). (c) Parasitism, hypothetical interaction where AMF species can parasitize other AMF species by penetrating the cell wall and growing within the invaded protoplasmic space. Lower panel: (d) Non‐interacting, where hyphae grow past one another without interaction. (e) Pre‐fusion incompatibility, in which tips swell upon contact but subsequently retract without fusion. (f) Post‐fusion incompatibility, where initial fusion is followed by cytoplasmic retraction and the formation of septa to isolate the interacting compartments. (g) Perfect fusion, also known as anastomosis, involves successful fusion and cytoplasmic continuity between hyphae and theoretically could result in the creation of an AMF heterokaryotic (also referred to as AMF dikaryotic) strain. To this day, only five AMF heterokaryotic strains (out of hundreds examined) have been identified globally, all belonging to the species *Rhizophagus irregularis*. (h) Lastly, the new suggested category of post‐fusion protoplasmic competitive exclusion. A hypothetical interaction where protoplasmic elements compete and can be eliminated across interconnected AMF mycelia. Different species are distinguished by the colour of their cell walls (black or purple), while different genomes are indicated by the colour of their nuclei (blue or red). Image courtesy of Sean Christopher Maston.

In addition to investing in expanding our trait databases by considering phenotypic complexity (Antunes *et al*., 2025; Marín & Wade, 2025), we also need to better understand the fungal interactions that take place within and outside of the host at the cellular level. Indirect evidence of mycelial competition between species (Hepper *et al*., 1988) suggests that AMF networks do not operate in isolation; rather, physical interactions are likely to occur both within and between species (Fig. 4). The exact outcomes when mycelia of different AMF species (interspecifically) come into physical proximity or contact remain poorly understood. While it is often assumed that genetically distinct species avoid direct interaction or fusion, empirical evidence to support this is limited, and actual behaviour may deviate significantly from this expectation. There are reports of AMF spores, typically from larger species such as those in the Gigasporaceae, being colonized by other AMF taxa, sometimes containing between one and five additional spore morphs within (Koske, 1984). Although Koske (1984) noted no clear signs of parasitism and suggested that colonization occurred post‐mortem of the host spore, definitive evidence is lacking, since spores were extracted via the standard wet sieving method (Pacioni, 1992) which captures only a single timepoint and does not allow temporal resolution of interspecific dynamics. It is possible that mycoparasitic mechanisms (physical or enzymatic) have evolved in AMF similarly to those observed in other fungi (De Jaeger *et al*., 2010). For example, *Trichoderma harzianum* employs a ‘Trojan horse‐like’ strategy, breaking into the ERM of various AMF through localized wall breaking and degradation by targeting mainly the electron dense components of the wall and not the chitin (e.g. *G. margarita*, *G. gigantea*, and *R. irregularis*) and eventually invading the plant host by breaching the IRM (De Jaeger *et al*., 2010; Lace *et al*., 2015). This behaviour aligns perfectly with the observations of *Lim et al*. (1983), who observed AMF‐like hyphae (unknown taxon) growing inside hyphae of *Rhizophagus fasciculatus* in *Trifolium repens* roots, although in this case, it was hypothesized to be a secondary structure of the fungus itself, rather than as a separate invading AMF. The increasing availability of whole genomes across AMF phylogeny should enable *in silico* analysis to detect evidence of such mechanisms.

Another possibility is that so‐called ‘forced interactions’ between species are driven by stress or disturbance, such as mechanical damage via soil disruption. In other fungal groups (e.g. *Neurospora*), introgression occurs under such conditions, where hybridization between diverse species leads to the exchange of genetic material, often producing morphologically novel but sterile spores due to chromosomal incompatibilities (Sun *et al*., 2012). If similar events occur in AMF, we might observe hyphal fusion between genetically divergent species (e.g. during the healing of cytoplasmic leakage) leading to chimeric individuals. These could form spores with mixed or unstable nuclear content, potentially appearing morphologically hybrid but lacking reproductive potential and/or viability. Such dynamics could contribute to the observed unexplained variation/plasticity in naturally occurring AMF spore morphology and function. In other fungal systems, introgression can also lead to viable progenies, resulting in enhancing, impairing, or diversifying functionality, eventually contributing to speciation (Zhang *et al*., 2018). We should use the recent innovations in culturing (Paré *et al*., 2022), high‐throughput imaging (Klein *et al*., 2024; Bisot *et al*., 2025; Oyarte Galvez *et al*., 2025), microscopy (Cargill *et al*., 2025), and molecular techniques (Yildirir *et al*., 2021; Sperschneider *et al*., 2023) to examine interactions between species at the network and cell level across varying physicochemical conditions.

Intraspecifically, mycelial interactions have been better characterized (mainly for *R. irregularis* and *F. mosseae*). The type of interaction depends on the level of somatic compatibility (Barreto de Novais *et al*., 2017) and includes non‐interacting, pre‐fusion incompatibility, post‐fusion incompatibility, and perfect fusion, also known as anastomosis (Croll *et al*., 2009) (Fig. 4). Anastomosis, besides increasing network integration, in many filamentous fungi, permits the establishment of heterokaryons, allowing genetically distinct nuclei to coexist within a shared cytoplasm. AMF strains have also been shown to exist as either homokaryons, that is, containing genetically similar nuclei, or heterokaryons (also referred to as dikaryons), that is, containing nuclei of two genetically divergent nucleotypes (Ropars *et al*., 2016; Kokkoris *et al*., 2021). The heterokaryotic condition appears functionally relevant, with such strains exhibiting enhanced growth and adaptability across host species (Serghi *et al*., 2021) at the expense of symbiotic function, with heterokaryons delivering lesser nutritional benefit to their hosts compared to homokaryotic strains (Terry *et al*., 2023). Although AMF heterokaryosis is hypothesized to arise from hyphal anastomosis between genetically distinct strains, stable dikaryon formation has not yet been demonstrated *in vitro*.

In other filamentous fungi within the Ascomycota and Basidiomycota, somatic compatibility is regulated by vegetative incompatibility (vic) or heterokaryon incompatibility (het) loci that mediate self/non‐self‐recognition and can trigger programmed cell death upon fusion of genetically dissimilar mycelia (Glass & Kaneko, 2003). This genetic system forms the basis of vegetative compatibility groups, which restrict cytoplasmic exchange and act as a defence system against transmission of viruses (Cortesi *et al*., 2001) or aggressive nuclei (Debets & Griffiths, 1998). Yet, despite numerous experiments designed to observe crossings between strains or species (Giovannetti *et al*., 2001; Croll *et al*., 2009; Purin & Morton, 2011; Barreto de Novais *et al*., 2013, 2017; de la Providencia *et al*., 2013), the molecular mechanisms underlying somatic compatibility in this group remain unresolved.

**Fig. 5:**
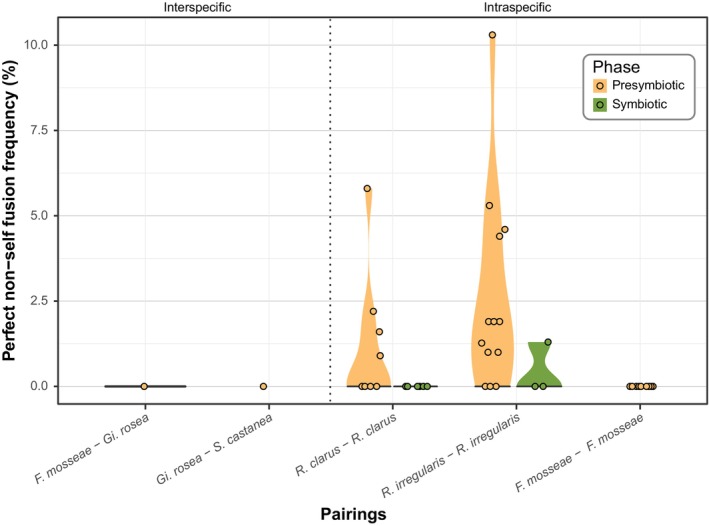
Interspecific and intraspecific perfect hyphal fusion compatibility in arbuscular mycorrhizal fungi (AMF). Violin plots show the distribution of perfect fusion frequency (%) between AMF pairings observed during distinct mycelial growth phases (presymbiotic (orange) vs. symbiotic (green)). Data were extracted from five key studies (Giovannetti *et al*., 1999, 2003; Croll *et al*., 2009; de la Providencia *et al*., 2013; Purin & Morton, 2013). Perfect fusions between different species have never been observed. Similarly, there is no difference between geographically distant *F. mosseae* strains. By contrast, perfect fusions have been documented between genetically distinct strains of *Rhizophagus clarus* and *R. irregularis* which can range from 0 to *c*. 6% to 0 to *c*. 10% of contact points, respectively. Notably, in *R. irregularis, a* higher frequency of perfect fusions occurs during the presymbiotic (*c*. 10% frequency) compared to the symbiotic phase (*c*. 1.3% frequency), but only in the presymbiotic phase in *R. clarus*, indicating distinct mycelial function in terms of hyphal compatibility between these two phases. *R. clarus* presents a good target species for detecting AMF heterokaryons beyond *R. irregularis*. Each violin plot represents the density and spread of the data for a given pair‐phase combination, with wider sections indicating a higher concentration of data points (circles). Overlaid circles represent individual observations, where each observation (*n*) is the mean of paired replicates within each study. For details on strain identity between these pairings, see Supporting Information Table S2. The figure was generated using Rstudio.

Equally surprising is that although perfect fusions have been observed between genetically distinct AMF strains during the presymbiotic (Giovannetti *et al*., 1999, 2003; Croll *et al*., 2009; de la Providencia *et al*., 2013; Purin & Morton, 2013) and symbiotic phase (de la Providencia *et al*., 2013; Purin & Morton, 2013) (Fig. 5), novel, stable heterokaryons have never been produced. This, in combination with the overall low abundance of heterokaryotic AMF globally (Kokkoris *et al*., 2021), raises questions about the contribution and function of anastomosis to this process. This disparity could be due to the two following reasons that have never been considered in AMF, but commonly occur in other fungal groups.

1. Following anastomosis, post‐fusion protoplasmic competitive exclusion may occur, resulting in the complete elimination of one of the two nucleotypes. In several filamentous fungi, fusion between genetically distinct strains can trigger strong antagonistic interactions at the protoplasmic level, where one nucleus type outcompetes or eliminates the other (Rayner, 1991). Similar protoplasmic competition could occur in AMF, resulting in the rapid loss of one nucleotype post‐fusion, without any external morphological sign and despite the presence of nuclei in anastomosing hyphae. The elimination of one nucleotype may be rapid, subtle, and cytologically difficult to capture, especially given the multinucleate and coenocytic nature of AMF hyphae and the lack of temporally resolved nuclear dynamics.
2. AMF may actively segregate their hyphae (via septa) to retain their individuality. As seen in *Neurospora crassa*, if incompatibility is detected, septa segregate the connected region and the isolated region is subjected to cell death to prevent stable heterokaryon formation between non‐self‐individuals (Hartl *et al*., 1975; Saupe, 2000; Glass & Kaneko, 2003). If a similar mechanism operates in AMF, it could prevent the mixing of incompatible cytoplasm or nuclei, maintaining genetic individuality despite transient physical fusion. For this reason, mycelial level observations are necessary, focusing on examining septa formation and hyphal viability at zones where genetically distinct strains meet.

Briefly, mycelial interactions among AMF likely reflect a spectrum from cooperation to conflict, with outcomes shaped by phylogeny, mycelial compatibility, and environmental factors. In contrast to the widely adopted view, anastomosis might not always translate to benefit for the interacting strains. It is possible that fused strains could be subjected to intense protoplasmic competition, could get eliminated, could get infected with mycoviruses and other selfish genetic elements reducing their fitness, and in general could be subjected to many more interactions than previously expected.

## VI. Content and coordination

The emergent behaviour of the mycelium depends on a finely tuned interplay of intracellular processes. Within AMF mycelia, a full complement of organelles–including nuclei, lipids, mitochondria, Golgi bodies, vesicles, and vacuoles–is arranged dynamically. The historical account presented here is necessarily partial, as it has been recently summarized with an emphasis on crucial cytological information (Bonfante, 2018). We have also previously detailed AMF cell content and organization (Kokkoris *et al*., 2020; Cargill *et al*., 2025), and this will not be repeated here. Instead, in this section I will focus on how the spatiotemporal distribution of cellular elements across the hyphal network reveals key insights into intracellular transport, resource allocation, and coordinated behaviour. These dynamics are influenced by both established and underexplored processes such as cytoskeletal motors and hydrostatic pressure, gradient‐based movement, and electrical signalling. These mechanisms are often interlinked and could serve as important components of mycelial coordination in AMF.

Cytoplasmic flow, or cytoplasmic streaming, represents an active transport system that compensates for the slow diffusion rates over a large fungal network (Goldstein & van de Meent, 2015). Particles within the AMF cytoplasm have been observed averaging speeds of 20 μm s^−1^, with bursts of speeds up to 120 μm s^−1^. Bidirectional and dynamic patterns likely reflect the need to transport resources and information in two opposing directions simultaneously (hyphal tips vs colonized roots) (Oyarte Galvez *et al*., 2025). Interestingly, some organelles can move independently of bulk cytoplasmic flow or remain anchored in specific zones (Bago *et al*., 1998a,c; Kokkoris *et al*., 2020), pointing to multiple diverse mechanisms acting synchronously, including pressure gradients (Lew, 2011) and motor‐driven transport along the cytoskeleton (Roper *et al*., 2015). While these mechanisms have been examined in detail in other fungal systems, they have been rarely examined in AMF (e.g. in *F. mosseae*) (Astrom *et al*., 1994). How exactly the cytoskeleton is arranged across the mycelium and to what extent it contributes to cytoplasmic and mycelial behaviour remains to be seen (Cargill *et al*., 2025).

In filamentous fungi, hydrostatic cell pressure (turgor pressure) is generated by solute accumulation and is a key force driving hyphal expansion and cytoplasmic flow. This process is linked to localized cell membrane and cell wall alterations, causing Ca^2+^ sequestration behind the tip, altering pressure locally (Lew, 2011). The presence of hyphal tips in both ERM and IRM points towards opposing pressure gradients found intraradically and extraradically, pulling cytoplasm apically but in opposing directions, possibly contributing to the intricate bidirectional flows observed in AMF (Cargill *et al*., 2025; Oyarte Galvez *et al*., 2025). The expected pressure differences at hyphal tips are supported in part by pH gradients observed intracellularly in *Gi. margarita* and *R. irregularis*, where tip pH (*c*. 6.7 at 0–2 μm) rises to *c*. 7.0 just behind the apex (*c*. 9.5 μm), then gradually decreases to *c*. 6.6 over the next 250 μm (Jolicoeur *et al*., 1998). How such fine pressure differences can be maintained across a coenocytic cell, though, is unknown, but the presence of tubular vacuolar systems is seen in *Gi. margarita* (Uetake *et al*., 2002) implies potential for longitudinal turgor pressure regulation across the mycelium.

The dynamic cytoplasmic flow could result in macropolarity across the mycelium, which is expected to affect other cellular properties such as ion transport, which in turn could enhance osmotic gradients and generate electrical currents. Electrophysiological studies in the ERM of *Gi. margarita*, *S. calospora*, and *F. coronatus* revealed that external hyphae and germ tubes maintain transmembrane electric potentials of *c*. −40 mV, sufficient to drive high‐affinity phosphate uptake. These values can rapidly drop in response to exogenous signals (e.g. addition of strigolactones), indicating that the first layer of response is linked to electrophysiology before gene activation (Ayling *et al*., 2000). Furthermore, reversible depolarization in response to external K^+^ suggests that AMF possess active control over ion permeability, supporting the presence of functional membrane ion channels (Ayling *et al*., 2000). This points towards a multi‐modal sensory system enabling rapid reaction to environmental cues. Incorporating biophysical assays, electrophysiology, functional genomic, and advanced microscopy (Knight *et al*., 1993) will be helpful in testing how these processes act on AMF and how they contribute to intracellular transport and mycelial coordination.

An added layer of complexity emerges when considering how ionic gradients interlink AMF to their bacterial endosymbionts, such as the endosymbiotic bacterium *Candidatus* Glomeribacter gigasporarum inhabiting *G. margarita* cells (Salvioli *et al*., 2016). These endobacteria enhance sporulation, elevate ATP production, and activate oxidative stress pathways, particularly during the presymbiotic phase. Critically, these endosymbionts reduce the fungus's basal intracellular Ca^2+^ levels while heightening sensitivity to strigolactones, suggesting that internal symbionts may influence membrane excitability and Ca^2+^ signalling cascades affecting environmental perception (Salvioli *et al*., 2016). More recently, it was also shown that the soil‐dwelling bacterium *Bacillus velezensis* can affect the AMF cell by increasing cytoplasmic flows via the production of surfactin (Anckaert *et al*., 2024). While the exact mechanisms of action were not examined, it is possible that surfactin is also linked to cation dynamics since it has been shown to induce voltage‐independent and cation‐selective channels in membranes (Sheppard *et al*., 1991). This highlights once again that AMF mycelia are shaped collectively by a combination of internal processes, external signals, and interactions with plant hosts, surrounding organisms, and endosymbionts (Richter *et al*., 2025), reinforcing the view of the mycelium as a responsive, multi‐organismal system.

## VII. Future directions

AMF mycelium is a dynamic, multifunctional structure central to AM symbiosis, yet still incompletely understood. Despite decades of research, gaps remain in our understanding of how AMF mycelial networks are assembled, maintained, and coordinated in space and time and across taxa. What molecular and biochemical mechanisms control the differentiation of core structures and how conserved (or variable) are these across the AMF phylogeny? How do environmental and genetic factors shape the balance between extraradical and intraradical investment, and can we develop standardized metrics to meaningfully compare these strategies across taxa? How do AMF regulate septation, protoplasmic retraction and advancement, and recovery at the cellular level, and to what extent do these responses vary per lineage? What detoxification mechanisms act in AMF mycelia and how can we better define overall fungal fitness? Do genetically distinct networks compete, cooperate, or even parasitize one another, and what mechanisms govern somatic compatibility and fusion outcomes? How are nuclei and other organelles organized and moved across large coenocytic cells, and what role do cytoskeletal, turgor pressure, and electrophysiological systems play in long‐distance coordination? These questions reflect a broader need to shift from descriptive observations to mechanistic understanding. Addressing them requires interdisciplinary approaches and the development of new tools and frameworks that link cellular spatiotemporal organization to emergent behaviour, fungal fitness, and ecological impact. As new imaging, molecular, and culturing technologies emerge, we are now getting equipped to examine the AMF mycelium at a higher level of complexity.

## Competing interests

None declared.

## Disclaimer

The New Phytologist Foundation remains neutral with regard to jurisdictional claims in maps and in any institutional affiliations.

## Supporting information


**Table S1** Advantages and limitations of biomass quantification methods in arbuscular mycorrhizal fungi.
**Table S2** Interspecific and intraspecific perfect hyphal fusion compatibility in arbuscular mycorrhizal (AM) fungi.

**Video S1** Cytoplasmic continuity between the extraradical and intraradical mycelium of the arbuscular mycorrhizal fungus *Rhizophagus irregularis* colonizing roots of *Helianthus tuberosus* in the SAP culturing system. Courtesy of Louis Paré.
Please note: Wiley is not responsible for the content or functionality of any Supporting Information supplied by the authors. Any queries (other than missing material) should be directed to the *New Phytologist* Central Office.
