# Supplementary material for: Mycelial dynamics in arbuscular mycorrhizal fungi
Source: New Phytol. 2025 Oct 26;249(2):691–713. doi: 10.1111/nph.70688 (PMC12712441; doi:10.1111/nph.70688)
Supplement: Supplementary file 1 — Table S1 Advantages and limitations of biomass quantification methods in arbuscular mycorrhizal fungi. Table S2 Interspecific and intraspecific perfect hyphal fusion compatibility in arbuscular mycorrhizal (AM) fungi. [file NPH-249-691-s001.pdf]

New Phytologist Supporting Information

Article title: Mycelial Dynamics in Arbuscular Mycorrhizal Fungi

Authors: Vasilis Kokkoris

Article acceptance date: 25 September 2025

Table S1. Advantages and limitations of biomass quantification methods in arbuscular mycorrhizal fungi

**Technique**	**Target / Focus**	**Strengths**	**Limitations**	**Best-use scenarios**
Hyphal length measurement	ERM = extraradical mycelium length (cm/g soil), IRM = intraradical colonization (%)	Widely used; provides basic estimates of fungal presence in soil/roots	Different units make ERM vs. IRM comparisons unsuitable; IRM % lacks info on density/intensity; ERM can be misidentified	Proof of colonization and in controlled* lab/greenhouse experiments assessing ERM length
Chitin- or ergosterol-based assays	General fungal cell wall / sterol biomarkers	Potential proxy for AMF biomass	Poor specificity as signals can include other fungi; unreliable in environmental samples	Useful in controlled* lab/greenhouse experiments
PLFA 16:1ω5	Phospholipid fatty acid biomarker	Potential proxy for AMF biomass	Non-specific – also found in bacteria; unreliable in environmental samples	Useful in controlled* lab/greenhouse experiments
NLFA 16:1ω5	Neutral lipid fatty acid	Can give meaningful biomass comparisons	Misestimates biomass when *Gigaspora* is present (low NLFA content)	Suitable for field surveys where *Gigaspora* is absent/rare
High-throughput imaging (IRM or ERM specific)	Actual length of ERM and number of arbuscules for the IRM	Allows temporal observations, accurate, high resolution	Designed for IRM or ERM separately, not both simultaneously; only for *in-vitro* cultures	Detailed time-course studies of either IRM or ERM dynamics
DNA quantification (qPCR, ddPCR)	Gene copy numbers (nuclear or mitochondrial)	Potentially most efficient upon optimization; can be phylogenetically tailored	Commonly used rDNA is multi-copy and does not correspond to biomass	Comparative studies across taxa/families when primers/probes are carefully validated; community-level quantification with calibration

* Experiments where the starting substrate is sterile

6 **Table S2.** Interspecific and intraspecific perfect hyphal fusion compatibility in arbuscular mycorrhizal (AM) fungi.

**Pairings**	**Strain Identity**	**Type of interaction**	**Phase**	**Perfect fusion frequency (non-self) (%)**	**References**
Rhizophagus irregularis - R. irregularis	DAOM197198-DAOM234328	Intraspecific	Presymbiotic	0	de la Providencia et al. (2013)
Rhizophagus irregularis - R. irregularis	DAOM197198-DAOM240415	Intraspecific	Presymbiotic	0	de la Providencia et al. (2013)
Rhizophagus irregularis - R. irregularis	DAOM240425-DAOM234328	Intraspecific	Presymbiotic	1,27	de la Providencia et al. (2013)
Rhizophagus irregularis - R. irregularis	A4-B3	Intraspecific	Presymbiotic	1,9	Croll et al. (2009)
Rhizophagus irregularis - R. irregularis	A4-C2	Intraspecific	Presymbiotic	4,6	Croll et al. (2009)
Rhizophagus irregularis - R. irregularis	A4-C3	Intraspecific	Presymbiotic	10,3	Croll et al. (2009)
Rhizophagus irregularis - R. irregularis	A4-D1	Intraspecific	Presymbiotic	0	Croll et al. (2009)
Rhizophagus irregularis - R. irregularis	C2-B3	Intraspecific	Presymbiotic	1,9	Croll et al. (2009)
Rhizophagus irregularis - R. irregularis	C2-C3	Intraspecific	Presymbiotic	5,3	Croll et al. (2009)
Rhizophagus irregularis - R. irregularis	C2-D1	Intraspecific	Presymbiotic	1,9	Croll et al. (2009)
Rhizophagus irregularis - R. irregularis	C3-B3	Intraspecific	Presymbiotic	1	Croll et al. (2009)
Rhizophagus irregularis - R. irregularis	C3-D1	Intraspecific	Presymbiotic	1	Croll et al. (2009)
Rhizophagus irregularis - R. irregularis	D1-B3	Intraspecific	Presymbiotic	4,4	Croll et al. (2009)
Funneliformis mosseae - F. mosseae	BEG12 - BEG 20	Intraspecific	Presymbiotic	0	Giovannetti et al., (1999)
Funneliformis mosseae - Gigaspora rosea	BEG12 - BEG 9	Interspecific	Presymbiotic	0	Giovannetti et al., (1999)
Funneliformis mosseae - Gigaspora rosea	BEG20 - BEG 9	Interspecific	Presymbiotic	0	Giovannetti et al., (1999)
Gigaspora rosea - Scutelospora castanea	BEG9 - BEG 1	Interspecific	Presymbiotic	0	Giovannetti et al., (1999)
Funneliformis mosseae - F. mosseae	AZ225C-IN101C	Intraspecific	Presymbiotic	0	Giovannetti et al. (2003)
Funneliformis mosseae - F. mosseae	AZ225C-BEG69	Intraspecific	Presymbiotic	0	Giovannetti et al. (2003)
Funneliformis mosseae - F. mosseae	AZ225C-BEG69	Intraspecific	Presymbiotic	0	Giovannetti et al. (2003)
Funneliformis mosseae - F. mosseae	AZ225C-IMA1	Intraspecific	Presymbiotic	0	Giovannetti et al. (2003)
Funneliformis mosseae - F. mosseae	IMA1-BEG25	Intraspecific	Presymbiotic	0	Giovannetti et al. (2003)
Funneliformis mosseae - F. mosseae	IMA1-IN101C	Intraspecific	Presymbiotic	0	Giovannetti et al. (2003)
Funneliformis mosseae - F. mosseae	IN101C-SY710	Intraspecific	Presymbiotic	0	Giovannetti et al. (2003)
Funneliformis mosseae - F. mosseae	IN101C-BEG25	Intraspecific	Presymbiotic	0	Giovannetti et al. (2003)
Funneliformis mosseae - F. mosseae	AZ225C-SY710	Intraspecific	Presymbiotic	0	Giovannetti et al. (2003)
Funneliformis mosseae - F. mosseae	IMA1-SY710	Intraspecific	Presymbiotic	0	Giovannetti et al. (2003)
Funneliformis mosseae - F. mosseae	BEG69-IMA1	Intraspecific	Presymbiotic	0	Giovannetti et al. (2003)

Funneliformis mosseae - F. mosseae	BEG69-BEG25	Intraspecific	Presymbiotic	0	Giovannetti et al. (2003)
Funneliformis mosseae - F. mosseae	BEG69-IN101C	Intraspecific	Presymbiotic	0	Giovannetti et al. (2003)
Funneliformis mosseae - F. mosseae	BEG69-SY710	Intraspecific	Presymbiotic	0	Giovannetti et al. (2003)
Rhizophagus clarus - R. clarus	6AmA#2 -1UnC#7	Intraspecific	Presymbiotic	0	Purin & Morton (2013)
Rhizophagus clarus - R. clarus	6AmA#2-CRwest#8	Intraspecific	Presymbiotic	0	Purin & Morton (2013)
Rhizophagus clarus - R. clarus	1UnC#7-CRwest#8	Intraspecific	Presymbiotic	0	Purin & Morton (2013)
Rhizophagus clarus - R. clarus	1UnC#5-1UnC#7	Intraspecific	Presymbiotic	5,8	Purin & Morton (2013)
Rhizophagus clarus - R. clarus	1UnC#7-WV310#5	Intraspecific	Presymbiotic	0	Purin & Morton (2013)
Rhizophagus clarus - R. clarus	1UnC#7-WV123A#6	Intraspecific	Presymbiotic	0	Purin & Morton (2013)
Rhizophagus clarus - R. clarus	1UnC#7-WV123A#7	Intraspecific	Presymbiotic	0	Purin & Morton (2013)
Rhizophagus clarus - R. clarus	WV310#5-WV123A#6	Intraspecific	Presymbiotic	0,9	Purin & Morton (2013)
Rhizophagus clarus - R. clarus	WV123A#7-WV123A#6	Intraspecific	Presymbiotic	1,6	Purin & Morton (2013)
Rhizophagus clarus - R. clarus	WV123A#7-WV310#5	Intraspecific	Presymbiotic	2,2	Purin & Morton (2013)
Rhizophagus irregularis - R. irregularis	DAOM197198-DAOM234328	Intraspecific	Symbiotic	0	de la Providencia et al. (2013)
Rhizophagus irregularis - R. irregularis	DAOM197198-DAOM240415	Intraspecific	Symbiotic	0	de la Providencia et al. (2013)
Rhizophagus irregularis - R. irregularis	DAOM240425-DAOM234328	Intraspecific	Symbiotic	1,3	de la Providencia et al. (2013)
Rhizophagus clarus - R. clarus	6AmA#2-1UnC#7	Intraspecific	Symbiotic	0	Purin & Morton (2013)
Rhizophagus clarus - R. clarus	6AmA#2-CRwest#8	Intraspecific	Symbiotic	0	Purin & Morton (2013)
Rhizophagus clarus - R. clarus	1UnC#7-CRwest#8	Intraspecific	Symbiotic	0	Purin & Morton (2013)
Rhizophagus clarus - R. clarus	1UnC#5-1UnC#7	Intraspecific	Symbiotic	0	Purin & Morton (2013)
Rhizophagus clarus - R. clarus	1UnC#7-WV310#5	Intraspecific	Symbiotic	0	Purin & Morton (2013)
Rhizophagus clarus - R. clarus	1UnC#7-WV123A#6	Intraspecific	Symbiotic	0	Purin & Morton (2013)
Rhizophagus clarus - R. clarus	1UnC#7-WV123A#7	Intraspecific	Symbiotic	0	Purin & Morton (2013)
Rhizophagus clarus - R. clarus	WV310#5-WV123A#6	Intraspecific	Symbiotic	0	Purin & Morton (2013)
Rhizophagus clarus - R. clarus	WV123A#7-WV123A#6	Intraspecific	Symbiotic	0	Purin & Morton (2013)
Rhizophagus clarus - R. clarus	WV123A#7-WV310#5	Intraspecific	Symbiotic	0	Purin & Morton (2013)

References

Croll D, Giovannetti M, Koch AM, Sbrana C, Ehinger M, Lammers PJ, Sanders IR. 2009. Nonself vegetative fusion and genetic exchange in the arbuscular mycorrhizal fungus *Glomus intraradices*. *New Phytologist* 181: 924–937.

Giovannetti M, Azzolini D, Citernesi AS. 1999. Anastomosis Formation and Nuclear and Protoplasmic Exchange in Arbuscular Mycorrhizal Fungi. *Applied and Environmental Microbiology* 65: 5571–5575.

Giovannetti M, Sbrana C, Strani P, Agnolucci M, Rinaudo V, Avio L. 2003. Genetic Diversity of Isolates of Glomus mosseae from Different Geographic Areas Detected by Vegetative Compatibility Testing and Biochemical and Molecular Analysis Genetic Diversity of Isolates of Glomus mosseae from Different Geographic Areas Detected by. *Applied and Environmental Microbiology* 69: 616–624.

de la Providencia IE, Nadimi M, Beaudet D, Rodriguez Morales G, Hijri M. 2013. Detection of a transient mitochondrial DNA heteroplasmy in the progeny of crossed genetically divergent isolates of arbuscular mycorrhizal fungi. *New Phytologist* 200: 211–221.

Purin S, Morton JB. 2013. Anastomosis behavior differs between asymbiotic and symbiotic hyphae of *Rhizophagus clarus*. *Mycologia* 105: 589–602.
